# Long-term microwaving of denture base materials: effects on dimensional, color and translucency stability

**DOI:** 10.1590/1678-7757-2017-0536

**Published:** 2018-05-29

**Authors:** Nick POLYCHRONAKIS, Gregory POLYZOIS, Panagiotis LAGOUVARDOS, Andreas ANDREOPOULOS, Hien Chi NGO

**Affiliations:** 1 National and Kapodistrian University of Athens, School of Dentistry, Department of Prosthodontics, Athens, Greece.; 2 National and Kapodistrian University of Athens, School of Dentistry, Department of Operative Dentistry, Athens, Greece.; 3 National Technical University of Athens, School of Chemical Engineering, Department of Synthesis and Development of Industrial Processes, Athens, Greece.; 4 University of Sharjha, College of Dental Medicine, Department of Preventive and Restorative Dentistry, Sharjha, United Arab Emirates.

**Keywords:** Microwaves, Color, Translucency, Dimension, Denture bases

## Abstract

While the combined effect of microwave irradiation with cleansing solutions on denture base materials has been investigated, the effects of only using microwave irradiation and, more importantly, in a long-term basis, was not studied yet.

**Objective:**

The purpose of this study was to evaluate the effect of a long-term repeated microwaving on the dimensional, color and translucency stability of acrylic and polyamide denture base materials.

**Material and Methods:**

Thirty two specimens (32 mm x 10 mm x 2.5 mm) from polyamide (Valplast) and PMMA (Vertex Rapid Simplified) denture base materials were made. Eight specimens from each material were immersed in distilled water (control) and 8 were subjected to microwave exposure at 450 W for 3 minutes for a period simulating 224 days of daily disinfection. Linear dimension, color change (ΔE*) and translucency parameter (TP) were measured at baseline and after certain intervals up to 224 cycles of immersion, using a digital calliper and a portable colorimeter. The results were analysed using two-way repeated measures ANOVA to estimate possible differences among predetermined cycles and material type. Regression analysis was also performed to estimate the trend of changes with time. Statistical evaluations performed at a significance level of 5%.

**Results:**

Data analysis showed significant changes in length at baseline with an increasing number of cycles (p<0.05) and a significant interaction of cycle-material (p<0.001). The ΔΕ* parameter was significantly higher with a higher number of cycles (p<0.001), but it did not vary between materials (p>0.05). TP decreased similarly in both materials following microwave action but in a significantly higher level for Valplast (p<0.001).

**Conclusions:**

The results indicated that long-term repeated microwaving affects linear dimensional, color and translucency changes of both materials. Differences between PMMA and polyamide material were noted only in dimension and translucency changes.

## Introduction

Contamination of removable prostheses with microorganisms (e.g. bacteria, fungi or viruses) turns them into sources of infection risk that may affect denture wearers and dental professionals. It is imperative that such prostheses be disinfected, at the dental surgery or laboratory, to reduce risks of cross-contamination and to comply with infection control guidelines^3^.

Several methods of disinfection have been suggested^3^ ^,^ ^5^ ^,^ ^28^, mainly including immersion of dentures in chemical solutions of sodium hypochlorite, alkaline glutaraldehyde, 4% chlorhexidine, chlorine dioxide, and denture cleansers such as alkaline peroxides, herbal and photodynamic therapy^1^ ^,^ ^11^ ^,^ ^13^ ^,^ ^15^. However, past studies have indicated that such solutions affect the physico-mechanical properties of the materials used to construct removable prostheses^19^ ^,^ ^25^. Furthermore, bleaching of dental base materials, unpleasant taste to patients and oral tissue reactions are some of the adverse effects of chemical disinfectants^15^. The use of such solutions has also been considered to be time-consuming^15^.

To overcome the above drawbacks, microwave energy was proposed as a simple, safe, low-cost and effective alternative method of disinfection^26^ ^-^ ^28^. Microwave irradiation of dentures is performed by either the wet (placed in a water bath) or dry disinfection method. The microwave energy ranges between 450-650 W for a period of 2 to 10 minutes. However, temperature developing during microwave disinfection may have a negative impact on polymer structure. The fact that water starts boiling after 90 seconds of irradiation^22^ and the appliance remains at this temperature until the end of the disinfection cycle may further enhance an acrylic resin polymerization reaction, which in turn may result in denture distortion^14^. Furthermore, it is well-documented that temperature levels exceeding 77°C may distort the base of the denture due to the release of internal stresses trapped within the material during the polymerization procedure^7^. In order to minimize undesirable side effects of exposure to excessive temperature during microwaving, some researchers have recommended adding alkaline peroxide to the plain water to reduce the time that the denture base is exposed to high temperatures^28^.

The effects of repeated microwave energy on physico-mechanical properties of denture base materials have been investigated in many studies^2^ ^,^ ^4^ ^,^ ^6^ ^,^ ^14^ ^,^ ^23^ ^,^ ^24^ ^,^ ^27^ ^,^ ^29^ ^,^ ^31^. However, the maximum number of disinfection cycles in the above studies was 36. Such number of cycles is considered as a short period of denture life in service according to the 5 year normal average life suggested by Dorner, et al.^8^ (2010).

Dimensional stability of dentures during processing and while in-service is of great importance for denture fit and patient satisfaction. The effects of microwave disinfection on the dimensional stability of denture base materials have been extensively studied, and some of them showed significant dimensional changes^2^ ^,^ ^14^ ^,^ ^24^ ^,^ ^27^ ^,^ ^29^ ^,^ ^31^, while others reported dimensional stability^4^ ^,^ ^6^.

Color is considered a significant parameter for the aesthetic appearance of dentures since color change acts as an indicator of material aging or damage^21^. Previous studies examined the effects of denture cleansers on the color stability of denture base materials^10^ ^,^ ^18^, but there is little information on the effect on color of microwave irradiation as a method of disinfection. Polychronakis, et al.^23^ (2015) revealed no significant changes either in PMMA color or polyamide denture base materials after a short cleansing period of 30 cycles, with a solution prepared by dissolving a Corega tablet in 200 mL of water and combined with microwave irradiation (450 W for 2 minutes).

Translucency is also an important property of denture aesthetics^30^. Denture base materials should have a color similar to normal soft tissues, but also a translucency that will allow the light to pass through and reflect back normal tissue shades for a more natural appearance. It is important that both color and translucency of denture base materials be maintained throughout their clinical use^17^. However, there are no studies focusing on the translucency of denture base materials and how much it is affected by their color changes.

It is clear from the aforementioned that, although there have been reports concerning the effect of microwave irradiation associated with denture cleansing solutions, there are no studies published on the effects of only using microwave irradiation on denture base materials and their optical properties, such as color and translucency, especially when longer periods of disinfection are involved.

The purpose of this study was to investigate the potential effect of repeated microwave disinfection on the dimensional, color and translucency stability of acrylic and polyamide denture base materials. The null hypotheses tested were that no differences existed between the materials with respect to the effect of long-term microwaving on dimensional, color and translucency stability.

## Material and methods

### Material

**Figure 1:**
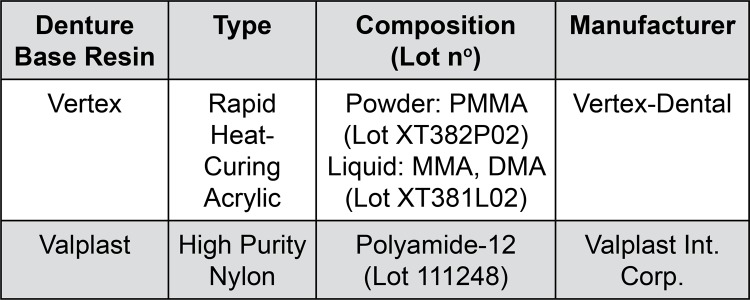
Type, composition and manufacturer of the materials used

For the purpose of this study, two denture base materials were selected. Material type, composition and manufacturer are shown in Figure 1.

### Specimen preparation

Sixteen identical machined stainless steel patterns (32 mm x 10 mm x 2.5 mm) were used for the purposes of the investment procedure. Sample size was decided in a priori estimation using G*Power software v.3.1.9.2 (Universität Kiel, Kiel, Germany), and an effect size (f) of 0.60, almost half the value found in a previous study^23^. Computations for the required sample size indicated a maximum total (for all four groups) size of 32, with actual power 0.962, 0.999, and 0.977 for between, within and within-between factors effects, respectively.

In the case of the polyamide specimens, a wax sprue was attached to the metallic pattern before investing, while in the PMMA’s case the conventional denture flasking technique was followed. The gap created after the boiling and removal of the patterns was filled with suitable material for the fabrication of specimens. Valplast was the polyamide material used and Vertex Rapid Simplified was the PMMA’s one. Before injecting polyamide into the gap, the material was heat-melted at 280°C for 11 minutes in a digital melting Valplast furnace. The flask was placed in the press for 3 minutes and then bench-cooled before opening. Following opening, the specimen was removed from the stone mold and the sprue was removed. PMMA specimens were fabricated according to the manufacturer’s instructions using a conventional pressure–pack technique and a 20 minutes water bath procedure at 100°C. After polymerisation, flasks were bench-cooled before specimens were removed from them.

Following deflasking, all specimens were stored in dry conditions. Initial finishing was performed with 600 grit, 800 grit and 1200 grit waterproof silicon carbide paper in an Ecomet III polishing equipment (Buehler Ltd, Evaston, III, USA) while polishing was performed using a high gloss agent (KMG, Candulor AG, Zurich, Switzerland) on a white cotton yam wheel polishing brush (Bur Dental, Guangzhoo, China). From each material, sixteen specimens were constructed, which were divided into two groups (n=8).

Group Ι (Control): specimens stored during the whole experimental period at room temperature (23±2°C) in a beaker filled with 200 ml of distilled water. The water changed at the same time intervals with experimental groups, following their measurement.

Group ΙΙ (Experimental): specimens were placed in a cup filled with 200 ml of distilled water and in a microwave device (Siemens-Electrogeräte HF1210, Siemens AG, Munich, Germany) operating at 450 W for 3, 7, 14, 28, 56, 84, 112, 140, 168, 196, and 224 cycles of 3 minutes^28^ each, simulating the effect of daily cleansing for almost 7.5 months.

The cycles were repeated until the number of cycles for the measurement was reached, and with a new water bath of 23±2°C for every new cycle. Specimens of both groups were immersed in distilled water and hanged in a small wooden rod fixed over the beaker using a nylon fishing line.

### Measurements

Measurements were taken: 1) immediately after specimens polishing (0dry); 2) after their immersion in distilled water for 48 hours (0wet); 3) after 3, 7, 14, 28, 56, 84, 112, 140, 168, 196, and 224 cycles in a microwave oven, or after 9, 21, 42, 84, 168, 252, 336, 420, 504, 588 and 672 minutes in distilled water for the control group. All measurements were carried out by the same investigator.

### Linear dimensional change measurements

The length of each specimen was measured using a digital caliper calibrated to 0.03 mm (Mitutoyo Inc., Tokyo, Japan). Each measurement was repeated 3 times and the mean value was calculated and recorded.

### Color measurements

The primary color parameters (lightness-L*, red/green-a* and yellow/blue-b*) of all specimens in the CIELAB system were measured with a portable contact type colorimeter (Shade Eye NCC, Shofu Inc., Kyoto, Japan) with a measuring window of 3 mm in diameter, against a white (L*=93.26, a*=-0.61, b*=2.09) and a black background (L*=2.93, a*=0.38, b*=-0.34). Measurements were performed at a right angle with the surface of the specimen, at three different sites in the middle area of the specimen for obtaining a mean value. The instrument was initially calibrated following manufacturer’s instructions, and then once every 20 measurements. The secondary parameters (ΔL*, Δa*, Δb*) were also estimated, and color differences were based on equation [1].

ΔE*=[(ΔL*)2+(Δa*)2+(Δb*)2]12 [1]

### Translucency measurements

Translucency of the specimens was estimated by calculating the TP (Translucency Parameter) using the equation [2], where “B” and “W” refer to color tristimulus values against a black (B) and white (W) background, respectively.

TP=(L*B-L*W)2+(a*B-a*W)2+(b*B-b*W)212 [2]

### Optical interferometric profiling

A noncontact optical interferometric profilometer (Wyko NT1100, Veeco, Santa Barbara, CA, USA) was used to take 3-D surface images of the specimens at the end of immersion time, for qualitative analysis. The instrument was operated in the vertical scan image mode of the Myro lens (5x2 FOV) at 20.4x total magnification, 10 mm back scan length, 30 mm scanning length and a modulation length of 2. The values of the Ra variables were estimated by scanning one surface *per* specimen.

### Statistical analysis

Data collected from measurements of the specimens’ length, color coordinates and translucency parameter (TP) values during repeated cycles in water with or without microwave irradiation were analysed statistically, using the 2-way repeated measures ANOVA in order to estimate possible statistically significant differences among predetermined cycles. Regression analysis was also performed to estimate the trend of changes with time. The above statistical evaluations and power estimations were performed using IBM-SPSS v.22 (IBM Corp. Armonk, NY, USA), at a significance level of 5%.

## Results

### Linear dimensional changes

Data obtained from changes in length of denture base materials at all predetermined number of cycles are presented in Table 1. Statistical Analysis (2-way repeated measures ANOVA) of the differences from baseline at all specified cycles revealed significant differences among material groups (p<0.001, power=1), while test of within-subjects effects showed significant differences among cycle groups (p<0.001, power=1) and a significant material-cycle interaction (p<0.001, power=1).

**Table 1:** Length difference (ΔLen) in mm with standard deviation (±) of all groups at all cycles

Material	3C	7C	14C	28C	56C	112C	224C
VALc	0.001±0.018	0.021±0.024	0.002±0.012	0.021±0.011	0.041±0.014	0.027±0.014	0.030±0.013
VALm	-0.087±0.025	-0.090±0.032	-0.0937±0.030	-0.055±0.029	-0.0475±0.032	-0.0537±0.029	-0.030±0.032
VERc	0.010+0.027	0.016+0.017	0.031+0.012	0.041+0.019	0.040+0.013	0.031+0.017	0.025+0.009
VERm	-0.074±0.025	-0.129±0.032	-0.180±0.031	-0.277±0.035	-0.327±0.034	-0.354±0.048	-0.349±0.050

Note: VALc= Valplast Control; VALm=Valplast Microwave; VERc=Vertex Control; VERm=Vertex Microwave

**Figure 2:**
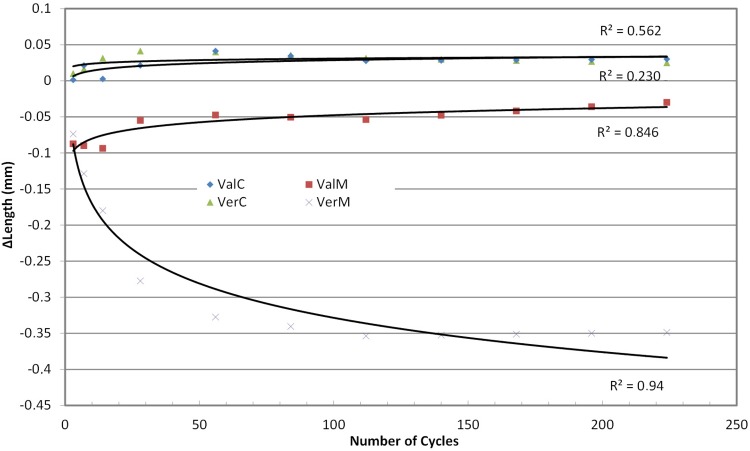
Trend lines for changes in ΔLength (mm)

The specimens’ length changed since the first cycles (Figure 2). The change was positive (ΔLength increased) for the control groups and remained stable for the rest of the cycles, but for the microwaved groups the change was negative (ΔLength decreased) and became more negative for the Vertex material and less negative for the Valplast material (Figure 2). Trend analysis on the above values indicated a rather logarithmic regression line of change along the number of cycles, and a much higher coefficient of determination (R^2^) for the microwaved group than in the control group, for both materials (Table 2).

**Table 2:** Line fitting based on R2 in Trend analysis for each investigated parameters

Material	ΔLen		ΔΕ*		ΔL*		Δa*		Δb*		ΔΤP	
	linear	log	linear	log	linear	log	linear	log	linear	log	linear	Log
VALc	0.302	0.563	0.019	0.204	0.151	0.476	0.238	0.426	0.124	0.272	0.085	0.006
VALm	0.738	0.847	0.972	0.778	0.130	0.297	0.795	0.778	0.887	0.783	0.692	0.941
VERc	0.007	0.230	0.642	0.850	0.656	0.845	0.237	0.237	0.889	0.740	0.425	0.202
VERm	0.600	0.940	0.408	0.734	0.461	0.838	0.240	0.240	0.426	0.402	0.793	0.886

Note: VALc= Valplast Control; VALm=Valplast Microwave; VERc=Vertex Control; VERm=Vertex Microwave

### Color changes

**Figure 3:**
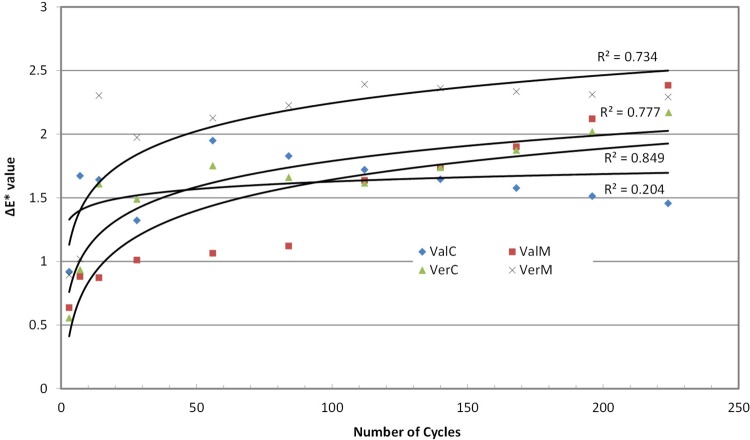
Trend lines for changes in ΔΕ* values

**Figure 4:**
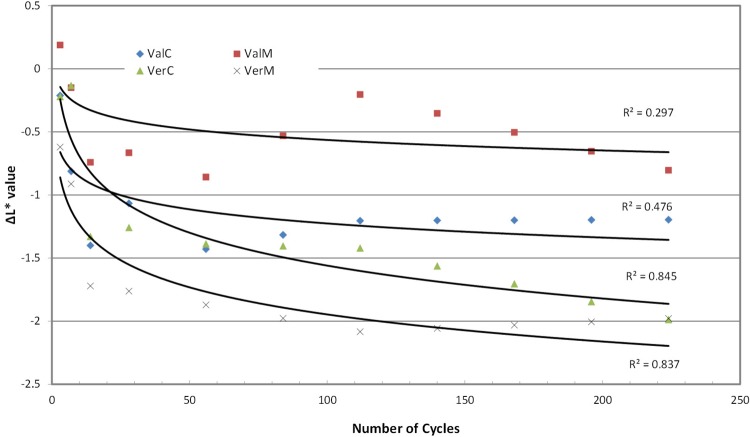
Trend lines for changes in ΔL* values

**Figure 5:**
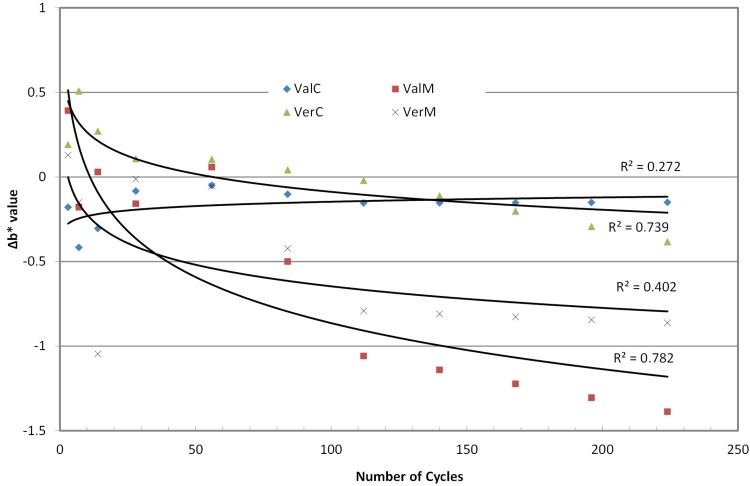
Trend lines for changes in Δa* values

**Figure 6:**
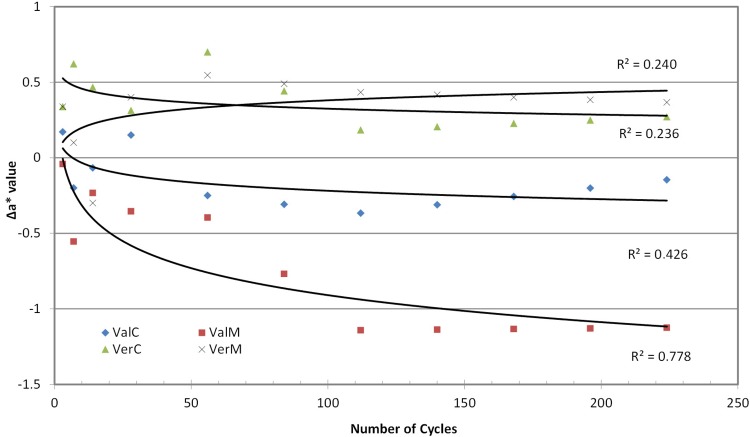
Trend lines for changes in Δb* values

Measurements of the color parameters ΔL*, Δa* and Δb* were used to calculate color differences (ΔΕ*) at baseline at the predetermined number of cycles (Figures 3 to 6). ΔE* parameter increased with the cycles up to 2.0-2.5 units at the end of the experiment, with the microwaved groups presenting the highest values. Statistical analysis (2-way repeated measures ANOVA) for ΔΕ* indicated no differences among material groups (p*=*0.063, power=1), but there were significant differences among cycle groups (p<0.001, power=1) and material-cycle interaction (p<0.001, power=0.997). Trend analysis also indicated a logarithmic regression line of change with the number of cycles (Table 2) with a greater R^2^ for Vertex Control, Vertex Microwaved and Valplast Control. These changes in ΔE* are the result of changes in L*, a* and b* parameters, which are shown in Figures 4-6. Most of the changes were negative, indicating a decrease when the number of cycles was increased. L* in Vertex, a* in Valplast and b* in both materials were decreased by the microwave action, as trend lines and R^2^ values indicate in these figures.

### Translucency changes

**Figure 7:**
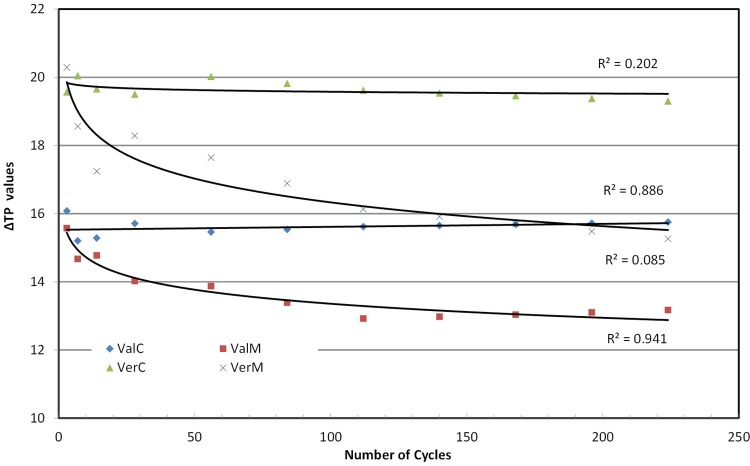
Trend lines for Translucency Parameter of the materials (TP units)

Calculations of TP for the groups at the specified cycle intervals, their difference from the translucency at the baseline are given in Figure 7 with their trend lines, and R^2^ following a logarithmic regression (Table 2). The figure shows that TP values for the Vertex material are initially higher than those of Valplast and remained the same for both materials of the control groups at all cycle intervals. TP values decreased for both materials when irradiated, and reached a decrease of 18.5%-22.5% after 224 cycles. Statistical analysis (2-way repeated measures ANOVA) indicated a significant difference between material groups (p<0.001, power=1), and among cycle groups (p<0.001, power=1), with material-cycle interaction (p<0.001, power=1). Trend analysis indicated a rather similar line for both materials (Table 2).

### Optical interferometric profiles

**Figure 8:**
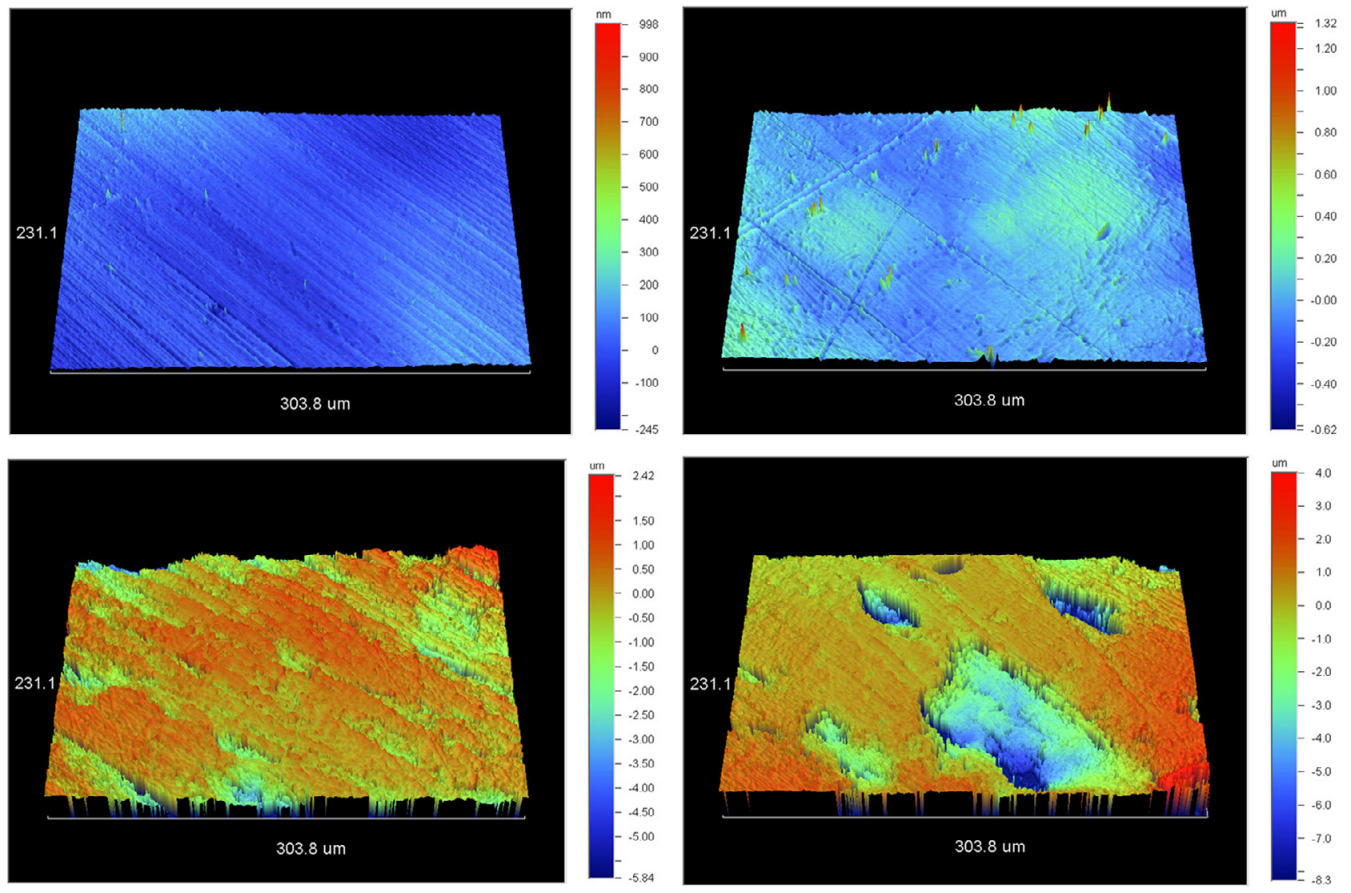
3-dimensional interactive display of a Vertex-control surface (upper left), Vertex-microwaved (upper right), Valplast-control (lower left) and Valplast-microwaved (lower right) at the end of immersion cycles

Representative profiles of PMMA and polyamide materials at the end of immersion cycles are given in Figure 8. The Ra parameter on the profile for polyamide material in the control group was found at around 497.46 nm, while that in the microwave group was found at around 953.89 nm. Ra values for PMMA were at 47.9 nm and 79.83 nm respectively. The above indicates a two times higher value for microwaved materials and ten times higher for the polyamide.

## Discussion

The hypothesis that no differences existed between denture base materials with respect to the effect of long-term microwaving on their dimensional and translucency stability was rejected, whereas accepted for the effects on color. This study’s results showed that microwave disinfection cycles did not significantly alter the color of both denture materials in relation to the control group, while significant differences were found between materials in dimensional and translucency.

In *in vitro* studies assessing dimensional changes of denture base materials, specimens are usually made of various shapes^2^ ^,^ ^27^ ^,^ ^31^, involving within changes numerous other factors besides processing and microwave disinfection. Palate shape, denture base thickness and teeth presence are some of the factors that lead to the final effect. In order to eliminate the influence of such factors, simple-shaped specimens instead of complex ones, such as denture base, were fabricated for the purpose of this study^12^.

### Length changes

Control groups showed only a slight expansion within water baths (less than 0.05 mm), probably due to water sorption by the hydrophilic acrylic resin, as a result of the polarity of its molecules^2^. However, both denture base materials under microwaving disinfection cycles exhibited a small shrinkage at first which later became greater for the Vertex (PMMA) and decreased slightly for the Valplast (polyamide).

The small initial shrinkage of both materials probably is the result of annealing, i.e. the effect caused by the release of existing internal stresses within the materials, caused by the direct or indirect action of irradiation. Its indirect action occurs possibly due to the temperature of the water bath rising above 90°C as early as the first minute in microwaving, while its direct action could be an improved diffusion at the surface of the materials, or an increase in the flux of vacancies within PMMA bead interfaces as in grain boundaries of the zirconia specimens^32^. The continuing shrinkage of PMMA can be explained by the diffusion of residual monomer^16^ to the active sites of the polymer chain^20^, leading to further polymerization and further shrinkage of the polymer^14^ or to the higher amount of grain boundaries existing in the PMMA material. In our study, it seems that the polyamide’s dimensional change ceased after 28 cycles and that of the PMMA’s ceased after 84 cycles, meaning that polyamides become dimensionally stable much sooner than PMMA.

Shrinkage effects of microwaving on PMMA denture base materials are similar to the effects in the studies of Gonçalves, et al.^14^ (2006) and Senna and Da Silva^27^ (2011), although in their studies the irradiation protocol was different (650 W for 6 minutes or 900 W for 3 minutes *per* cycle). In this study, the extend of shrinkage did not exceed -1.12%, which is in perfect agreement with a previous study of Polychronakis, Yannikakis and Zissis^24^ (2014) who found that PMMA showed linear shrinkage up to -1.16% following disinfection of seven 6 minutes cycles in a microwave oven at 650W. On the contrary, Basso, et al.^4^ (2010) reported no significant effect on dimensional stability. Alkhodary^2^ (2014) observed expansion in rectangular PMMA specimens after 7 or 28 cycles of microwave disinfection at 600 W or 700 W for 3 minutes. The controversy with our results may be related to different microwave disinfection protocols (power) and test specimens dimensions. Furthermore, Wagner and Pipko^31^ (2015) reported an expansion of PMMA after two exposures at 420 W microwave energy for 3 minutes, but the test specimens were denture bases and not rectangles. Another explanation for the difference between their and our results may be related to the type of materials and the polymerization cycle used for specimens’ fabrication, as well as to the methods applied for measuring dimensional changes.

### Color changes

Both resins tested in this study exhibited a statistically significant change of their initial color after microwave disinfection and reached the level of almost 2.5 units of ΔΕ* at the end of 224 cycles. However, this change was not considered important since it was below the clinically perceptible levels of 2.7^9^ (Figure 3). Similar changes in color for microwaving action were found by Polychronakis, et al.^23^ (2015) at 30 microwave disinfection cycles (0.67 for PMMA and 1.11 ΔΕ* units for polyamide).

Although color changes were not statistically significantly different between the two materials, they behaved differently. Looking at the graph of Figure 3, it is seen that the change for the Vertex was initially close to that of Valplast, but in two weeks reached the level of its total change and remained there until the end of 224 cycles (7.5 months). The change for Valplast was increasing with cycles, but the change was evident after 112 cycles (4 months) and continued to increase until the end of the experiment. This is why the trend line for Valplast was much steeper than Vertex’s. This model of color changes was also followed by the materials in the control baths. Valplast reached the level of 1.5 ΔΕ* at 7 cycles (one week) and remained there until the end of the experiment, while Vertex reached the same level after 14 days and, continuing to increase, it finally reached a higher level of change than Valplast’s. The change of the irradiated Valplast group was due to the decrease in a* and b* parameters, while that of irradiated Vertex was due to the decrease in L* and b*. The change in Valplast control group was mainly due to the decrease in L* and in Vertex to the decrease in L* with a small increase in a* (Figures 4-6). This means that polyamide becomes less red and yellow retaining its initial Lightness, while PMMA becomes less yellow and darker as the cycles in microwave oven are continued.

When extrapolating (forecasting) these effects to one year (at 364 cycles), only the Vertex-M reaches the level of 2.7 units due to its over than -2.0 units change in lightness. These changes are probably due to the loss of staining materials either by a direct effect of microwaves on the polyamide material or a greater diffusion of water molecules in the PMMA’s polymer matrix, some of which may additionally bind permanently to high energy sites of the polymer. This effect of water on PMMA material was also reported by Hong, et al.^18^ (2009), who found that the longer the immersion time, the greater the change in color.

### Translucency changes

Translucent denture base materials allow light to pass through them and reflect back the normal soft tissue’s shades, thus making the devices look more natural. Changes in translucency indicate either serious changes to the material’s mass, most likely irreversible, or an increase in their surface roughness, resulting in a greater diffusion and a lower reflectance of the light falling on their surfaces. In this study, TP values of Vertex were initially higher than those of Valplast (Figure 7). Within control baths, both materials retained their initial TP values (their loss was only 0.010%-0.012%), but in the microwave oven, TP values decreased significantly (18.7%-22.5%). Most probably, the decrease can be explained by the increase in roughness due to microwave irradiation on PMMA and polyamide material (Figure 8), as Polychronakis, et al.^23^ (2015) have already shown.

This study revealed a significant effect of the microwaving action on initial shrinkage, color and translucency of polyamide and PMMA denture base materials, which behaved differently. Most of the changes happened during the first 60-90 days, but they continue slowly thereafter.

Experimental design limitations of this work, such as testing of dimensional changes on rectangular specimens instead of actual dentures, make it difficult to compare the results of this *in vitro* study with those obtained under clinical circunstances.

Hence, it seems necessary to investigate the effect of long-term microwave disinfection on complete dentures alongside usage by patients, thus taking into consideration different influences on physico-mechanical properties and structure of denture base materials.

## Conclusions

Under the limitations of the present *in vitro* study, the following conclusions can be drawn:

Long-term microwaving increased the initial shrinkage of polyamide material slightly and decreased that of PMMA significantly. Initial color of both materials presented a similar change at about 2.0-2.5 ΔΕ* units, which resulted from a significant loss in L* for the PMMA and in a* and b* for the polyamide material. Finally, both materials behaved similarly and lost a significant amount (18.7%-22.5%) of their initial translucency under microwave irradiation in contrast to those in the control group, which lost only 0.01%.
